# Impact of the COVID-19 Pandemic on Health Check-ups in 2021 and 2022: A Nationwide Follow-up Survey of Healthcare Facilities in Japan Society of Ningen Dock

**DOI:** 10.31662/jmaj.2023-0126

**Published:** 2023-12-27

**Authors:** Satoko Yamaguchi, Tomofumi Atarashi, Akira Okada, Shigeru Nasu, Toshimasa Yamauchi, Yasuji Arase, Takao Aizawa, Masaomi Nangaku, Takashi Kadowaki

**Affiliations:** 1 Department of Prevention of Lifestyle-related Diseases, Graduate School of Medicine, The University of Tokyo, Tokyo, Japan; 2 Japan Society of Ningen Dock, Tokyo, Japan; 3 Medical Check-up Center, JA Hokkaido P.W.F.A.C. Obihiro-Kosei General Hospital, Hokkaido, Japan; 4 Hakuaikai Hospital, Fukuoka, Japan; 5 Department of Diabetes and Metabolism, Graduate School of Medicine, The University of Tokyo, Tokyo, Japan; 6 Health Management Center, Toranomon Hospital, Tokyo, Japan; 7 Aizawa Hospital, Nagano, Japan; 8 Division of Nephrology and Endocrinology, Graduate School of Medicine, The University of Tokyo, Tokyo, Japan; 9 Toranomon Hospital, Tokyo, Japan

**Keywords:** COVID-19 pandemic, health check-ups, cancer screenings, diabetes screenings

## Abstract

**Introduction:**

Preventive programs, including screenings for cancer and diabetes, were disrupted globally due to the coronavirus disease 2019 (COVID-19) pandemic in 2020. We previously conducted a nationwide survey to investigate the initial impact of the pandemic on health check-ups; however, the impact in the second and third years of the pandemic has not yet been elucidated. Here, we conducted a follow-up survey targeting healthcare facilities to evaluate the impact of the pandemic until the end of 2022.

**Methods:**

A questionnaire survey was conducted between December 15, 2022, and February 10, 2023, targeting member facilities of Japan Society of Ningen Dock. The survey consisted of two parts. Part I comprised a web-based questionnaire, in which the facilities were asked about their commitment to COVID-19-related care, precautions against COVID-19, and whether the pandemic had a negative financial impact on the management of health check-ups. In Part II, the facilities were asked about the number of examinees who underwent health check-ups between 2019 and 2022, the proportion of those who needed and adhered to follow-up visits, and the number of cancer cases found between 2019 and 2021.

**Results:**

Of the 1,343 eligible facilities, 885 participated (response rate: 65.9%). The observation that the number of people undergoing mandatory check-ups increased while those undergoing nonmandatory check-ups (e.g., cancer screenings by local governments) decreased in 2021, compared with that of 2019, persisted into 2022. Approximately 60% of the facilities reported a negative financial impact on the management of health check-ups, even in 2022.

**Conclusions:**

In 2022, the pandemic’s detrimental effects on health check-ups persisted.

## Introduction

The COVID-19 pandemic widely disrupted preventive programs such as cancer and diabetes screenings in 2020 ^(1), (2)^. To minimize the increase in avoidable deaths due to delayed screenings, a rapid catching up of missed screenings is desirable ^(3), (4)^. Reports evaluating the situation in the second year of the pandemic have only recently emerged. Studies from the US reported that, in 2021, screening prevalence for breast, cervical, and prostate cancer continued to be lower than prepandemic levels ^(5), (6)^. Another study from the UK reported that, even in 2021, diabetes screenings have not completely returned to prepandemic levels ^(2), (7)^.

In Japan, nationwide health check-up programs have been developed; annual health check-ups are mandatory for full-time employees under the Industrial Safety and Health Act ^(8)^, and all people aged 40-74 years are entitled to undergo annual Specific Health Check-ups aimed at screening high-risk populations for hypertension, diabetes, dyslipidemia, and metabolic syndromes ^(9)^. Additionally, local governments generally provide age-eligible cancer screenings for gastric, colorectal, lung, breast, and cervical cancer ^(10), (11)^.

In 2020, a decrease in the number of participants in population-based cancer screenings in Japan has been reported ^(12)^. We previously conducted a nationwide questionnaire survey to investigate the impact of the pandemic on health check-ups between 2020 and 2021 and reported that although the number of examinees undergoing mandatory check-ups recovered in 2021 from the decline in 2020, the number of those undergoing nonmandatory check-ups remained low in the same year ^(11)^.

The long-term impact of the COVID-19 pandemic on preventive programs is yet to be elucidated. Here, we report the findings of a nationwide follow-up survey of healthcare facilities to evaluate the impact of the pandemic between 2020 and 2022.

## Materials and Methods

### Questionnaire survey

Healthcare facilities were considered eligible if they were members of Japan Society of Ningen Dock and could respond by email as in the previous study ^(11)^. Japan Society of Ningen Dock has approximately 1,700 member facilities including hospitals and clinics across Japan, accounting for approximately half of all facilities conducting health check-ups ^(13)^, of which 1,343 facilities that could respond via email were considered eligible for the study.

The survey included two parts. Part I was a web-based questionnaire that participants could answer online. Facilities were asked to provide the following information: the average number of employees engaged in health check-ups per day; their commitment to COVID-19-related care, including whether the facilities were admitting patients with COVID-19, had outpatient fever clinics, or were providing COVID-19 vaccination services; precautions taken against COVID-19 to perform in-facility check-ups; recommendations made to employees as precautions against COVID-19; the number of employees who were absent from work for reasons related to COVID-19 in 2020, 2021, and 2022; and whether the COVID-19 pandemic had a negative financial impact on the management of health check-ups. In Part II, participants were asked to fill in a form and return it by email. This form collected information on the annual and monthly number of examinees who underwent health check-ups between 2019 and 2022 according to the type of check-ups (e.g, “Check-ups based on Industrial Safety and Health Act”, “Check-ups for prevention of lifestyle-related diseases”, “Cancer screenings by local governments”). Regarding screenings for cancer, hypertension, diabetes, and dyslipidemia between 2019 and 2021, these facilities were also asked to provide the number of examinees undergoing each screening, the proportion of examinees requiring follow-up visits, the proportion of examinees who attended follow-up visits, and the number of cancer cases found in each year. Emails containing URLs to the questionnaire and form were sent to the facilities on December 15, 2022, and responses were collected by February 10, 2023.

### Outcome measures

For each facility, the number of examinees undergoing check-ups in 2020, 2021, or 2022 was compared with that in 2019 (the preCOVID-19 year). The proportion of examinees requiring follow-up visits and adherence to follow-up visits, defined as the proportion of examinees who attended follow-up visits among those who were required to have them, were compared with those in 2019 for each facility as previously described ^(11)^.

Facilities were classified based on whether the COVID-19 pandemic had a negative financial impact on the management of health check-ups in 2022. The characteristics of the facilities with or without a negative financial impact in 2022 were compared.

### Statistical analysis

Medians and interquartile ranges (IQRs) were used to summarize continuous variables. The number of examinees undergoing check-ups in 2019, 2020, 2021, and 2022, the proportion of examinees requiring follow-up visits, and adherence to follow-up visits in 2019, 2020, and 2021 were compared for each facility using the Friedman test with Holm correction. The characteristics of the facilities by type or negative financial impact were compared using the chi-square test or Fisher’s exact test for categorical variables and the Mann-Whitney U test for continuous variables.

For multivariate logistic regression analyses to identify factors associated with a negative financial impact on the management of health check-ups, a negative financial impact in 2022 was defined as the dependent variable, and other facility characteristics were used as independent variables.

All statistical tests were two-sided, and *P* values of <0.05 were considered significant. All analyses were performed using R v4.1.1. (R Foundation for Statistical Computing, Vienna, Austria).

### Institutional review board approval

This study was approved by the institutional review board of the Graduate School of Medicine at the University of Tokyo (2018030NI) and was performed following the Declaration of Helsinki. The need for informed consent was waived because this was a facility-based survey that did not contain any personal information.

## Results

Among the 1,343 eligible facilities, 885 responded to the survey (overall response rate: 65.9%); 838 (62.4%) responded to Part I and 537 (40.0%) responded to Part II, among which 490 (36.5%) responded to both Parts I and II. Of the participating facilities, 543 (61.4%) were annexed to hospitals, 166 (18.8%) were annexed to clinics, and 176 (19.9%) were dedicated to healthcare check-ups. Regarding location, 334 (37.7%) facilities were located in ordinance-designated cities or one of the 23 special wards in Tokyo.

Of the 838 facilities that responded to Part I, 140 (16.7%), 237 (28.3%), 163 (19.5%), 86 (10.3%), 66 (7.9%), and 146 (17.4%) reported that there were 1-10, 11-20, 21-30, 31-40, 41-50, and >50 employees engaged in health check-ups per day, respectively.

Regarding their commitment to COVID-19-related care, 419 (50.0%) facilities accepted admission of patients with COVID-19, 518 (61.8%) had outpatient fever clinics, 650 (77.6%) provided COVID-19 vaccination services, and 149 (17.8%) had COVID-19 PCR centers. A negative financial impact on the management of health check-ups due to the COVID-19 pandemic was reported by 714 (85.2%) facilities. The proportions of facilities that had negative financial impacts in 2020, 2021, and 2022 were 80.5%, 73.0%, and 61.7%, respectively.

Of the 492 facilities whose number of check-ups between 2019 and 2022 were available, the median of the total number of check-ups in 2019 was 14,007 (IQR: 6,600-32,037), and the median percentage of mandatory “Check-ups based on Industrial Safety and Health Act” and “Check-ups for prevention of lifestyle-related diseases” was 34.0% (IQR: 18.0%-54.6%).

Table 1 shows facility characteristics by type. Facilities annexed to hospitals were less likely to be located in ordinance-designated cities/special wards in Tokyo and were more likely to be public institutions, have fewer employees and performed fewer check-ups in 2019, and have participated in COVID-19-related care than those dedicated to health check-ups or those annexed to clinics. In addition, facilities annexed to hospitals were more likely to have a negative financial impact in 2022. Consistently, the number of check-ups in facilities annexed to hospitals decreased significantly in 2022 compared with that in 2019, whereas that of facilities dedicated to check-ups or annexed to clinics in 2022 was comparable to those in 2019.

**Table 1.** Facility Characteristics by Type.

		Total (n = 838)	Dedicated to check-ups/Annexed to clinics (n = 320)	Annexed to hospitals (n = 518)	*P* value
Area^a^	Kanto	301 (35.9)	132 (41.3)	169 (32.6)	0.035
	Hokkaido	21 (2.5)	11 (3.4)	10 (1.9)	
	Tohoku	44 (5.3)	14 (4.4)	30 (5.8)	
	Chubu	157 (18.7)	45 (14.1)	112 (21.6)	
	Kansai	148 (17.7)	63 (19.7)	85 (16.4)	
	Chugoku	60 (7.2)	22 (6.9)	38 (7.3)	
	Shikoku	23 (2.7)	7 (2.2)	16 (3.1)	
	Kyushu	73 (8.7)	22 (6.9)	51 (9.8)	
	Okinawa	11 (1.3)	4 (1.3)	7 (1.4)	
Location of facilities	Ordinance-designated cities/special wards in Tokyo	310 (37.0)	172 (53.8)	138 (26.6)	<0.001
Public or private institutions	Public	190 (22.7)	17 (5.3)	173 (33.4)	<0.001
Number of employees	1-10	140 (16.7)	39 (12.2)	101 (19.5)	<0.001
	11-20	237 (28.3)	52 (16.3)	185 (35.7)
	21-30	163 (19.5)	54 (16.9)	109 (21.0)
	31-40	86 (10.3)	36 (11.3)	50 (9.7)
	41-50	66 (7.9)	38 (11.9)	28 (5.4)
	>50	146 (17.4)	101 (31.6)	45 (8.7)
Accepting admission of patients with COVID-19	Yes	419 (50.0)	11 (3.4)	408 (78.8)	<0.001
Outpatient fever clinic	Yes	518 (61.8)	64 (20.0)	454 (87.6)	<0.001
Providing COVID-19 vaccination services	Yes	650 (77.6)	183 (57.2)	467 (90.2)	<0.001
COVID-19 PCR center	Yes	149 (17.8)	18 (5.6)	131 (25.3)	<0.001
Providing help to other departments within the facility	Yes	482 (57.5)	139 (43.4)	343 (66.2)	<0.001
Providing help to other departments for COVID-19-related care	Yes	359 (42.8)	90 (28.1)	269 (51.9)	<0.001
Negative financial impact in 2020	Yes	675 (80.5)	253 (79.1)	422 (81.5)	0.346
Negative financial impact in 2021	Yes	612 (73.0)	224 (70.0)	388 (74.9)	0.098
Negative financial impact in 2022	Yes	517 (61.7)	176 (55.0)	341 (65.8)	0.001
Number of facilities^b^		(n = 492)	(n = 185)	(n = 307)	
Total number of check-ups in 2019	Median (IQR)	14007 (6600, 32037)	28518 (14645, 74869)	9833 (5113, 18466)	<0.001
	<10000	188 (38.2)	33 (17.8)	155 (50.5)	<0.001
	10000-19999	115 (23.4)	30 (16.2)	85 (27.7)	
	20000-39999	91 (18.5)	53 (28.6)	38 (12.4)	
	≥40000	98 (19.9)	69 (37.3)	29 (9.4)	
Percentage of mandatory check-ups^c^ in 2019	Median (IQR)	34.0 (18.0, 54.6)	31.6 (15.4, 60.0)	34.7 (19.0, 50.5)	0.622
	<34%	246 (50.0)	96 (51.9)	150 (48.9)	0.577
	≥34%	246 (50.0)	89 (48.1)	157 (51.1)	
Percent change in the number of check-ups in 2022 compared with 2019	Median (IQR)	−1.5 (−7.4, 6.0)	0.1 (−5.8, 9.1)	−2.5 (−8.9, 4.7)	0.001

^a^ Areas in Japan: Japan comprises the four main islands of Hokkaido, Honshu, Shikoku, and Kyushu. Honshu, the largest island, is divided into the Kanto (which includes the Greater Tokyo area), Tohoku, Chubu, Kansai, and Chugoku regions. Okinawa is the southernmost prefecture that includes the fifth largest island. ^b^ Number of facilities whose number of check-ups between 2019 and 2022 were available ^c^ Percentage of “Check-ups based on Industrial Safety and Health Act” and “Check-ups for prevention of lifestyle-related diseases”

### Changes in the annual number of health check-ups by type

**Figure 1.**
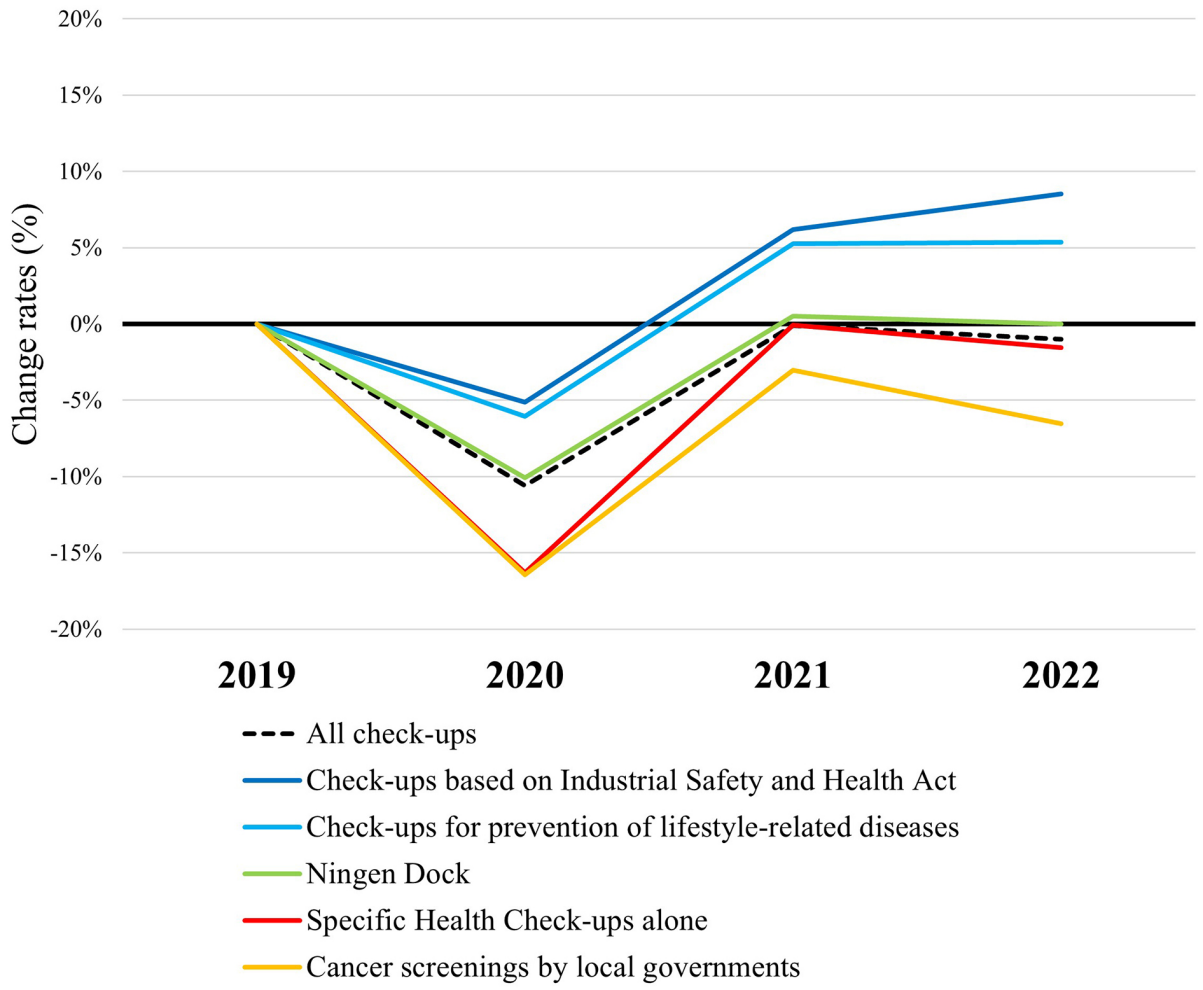
Change rates in the total overall annual numbers of check-ups and those by type compared with those in 2019 (n = 492).

The total overall annual number of examinees undergoing check-ups in 492 facilities between 2020 and 2022 was compared with those in 2019. The total overall of all check-ups changed by −10.6%, −0.1%, and −1.0% in 2020, 2021, and 2022, respectively (Figure 1), and the numbers in all types of check-ups decreased in 2020. As we previously reported, the extent of this decrease and the subsequent recovery in 2021 varied depending on the type of check-up ^(11)^. Notably, “Check-ups based on Industrial Safety and Health Act” are mandatory for full-time employees of all ages, and employers are obliged to offer them. “Check-ups for prevention of lifestyle-related diseases,” which contain additional tests such as some cancer screenings, are offered by insurers and can be conducted in place of “Check-ups based on Industrial Safety and Health Act.” Employers are obliged to report the results of exam rates for “Check-ups based on Industrial Safety and Health Act” to the Labor Standards Inspection Office. While the numbers of “Check-ups based on Industrial Safety and Health Act” and “Check-ups for prevention of lifestyle-related diseases” increased by 6.2% and 5.3% in 2021 and by 8.5% and 5.4% in 2022, respectively, the change rates for nonmandatory “Specific Health Check-ups alone” and “Cancer screenings by local governments” were −0.1% and −3.0% in 2021 and −1.6% and −6.5% in 2022, respectively. When the numbers of check-ups between 2020 and 2022 were compared with those in 2019 for each facility, those for all types of check-ups decreased in 2020; those for mandatory “Check-ups based on Industrial Safety and Health Act” and “Check-ups for prevention of lifestyle-related diseases” increased in 2021, whereas those for nonmandatory “Specific Health Check-ups alone” and “Cancer screenings by local governments” decreased significantly in 2021, as we previously reported ^(11)^. This tendency continued in 2022 (Table 2).

**Table 2.** Annual Number of Check-Ups between 2020 and 2022 Compared with 2019 for Each Facility.

	Mandatory^a^	2020 median (IQR)	*P* value^b^	2021 median (IQR)	*P* value^b^	2022 median (IQR)	*P* value^b^	Number of facilities^c^
All check-ups	-	−9.0 (−15.2, −4.0)	<0.001	−0.7 (−6.4, +4.5)	0.087	−1.5 (−7.4, +6.0)	0.022	492
Check-ups based on Industrial Safety and Health Act	Yes	−4.7 (−11.3, +1.5)	<0.001	+2.3 (−5.8, +10.8)	<0.001	+3.5 (−6.4, +16.1)	<0.001	441
Check-ups for prevention of lifestyle-related diseases	Yes	−5.3 (−13.1, +0.8)	<0.001	+4.9 (−2.3, +11.9)	<0.001	+5.3 (−3.6, +15.2)	<0.001	442
Comprehensive check-ups (Ningen Dock)^d^	Yes/No	−9.3 (−15.6, −3.6)	<0.001	−0.2 (−6.5, +6.3)	>0.99	−0.7 (−7.1, +9.0)	>0.99	479
Specific Health Check-ups alone^e^	No	−14.5 (−26.3, −4.5)	<0.001	−4.6 (−16.0, +9.2)	<0.001	−4.1 (−18.0, +12.6)	0.015	412
Cancer screenings by local governments	No	−13.1 (−24.3, −1.0)	<0.001	−3.1 (−16.0, +12.6)	0.009	−4.1 (−20.3, +20.3)	0.019	341
Other in-facility check-ups^f^	No	−10.9 (−22.8, +0.8)	<0.001	−3.6 (−18.2, +11.0)	0.002	−6.4 (−19.5, +12.2)	<0.001	455
Check-ups using mobile medical vehicles	Yes/No	−11.3 (−19.6, −5.2)	<0.001	−5.6 (−14.5, +0.3)	<0.001	−8.2 (−18.1, −1.1)	<0.001	156

^a^ Whether each type of check-up contains mandatory check-ups (yes), nonmandatory check-ups (no), or both mandatory and nonmandatory check-ups (yes/no) ^b^ *P* values comparing the numbers in 2020, 2021, and 2022 to those in 2019 using the Friedman test with Holm correction ^c^ For each type of check-up, the facilities whose annual number of examinees was zero in 2019 were excluded ^d^ Comprehensive check-ups (Ningen Dock) as defined by Japan Society of Ningen Dock ^e^ “Specific Health Check-ups alone” refers to Specific Health Check-ups without mandatory check-ups. All items in Specific Health Check-ups are included in the “Check-ups based on Industrial Safety and Health Act,” “Check-ups for prevention of lifestyle-related diseases,” and “Comprehensive check-ups Ningen Dock.” ^f^ Includes comprehensive check-ups (Ningen Dock) that do not fulfill the definition of Japan Society of Ningen Dock

Change rates in the total overall of the monthly number of check-ups and those by type between 2020 and 2022, compared with the same months in 2019, are shown in Supplementary Figure 1.

### Screenings for cancer, hypertension, diabetes, and dyslipidemia in 2021

**Figure 2.**
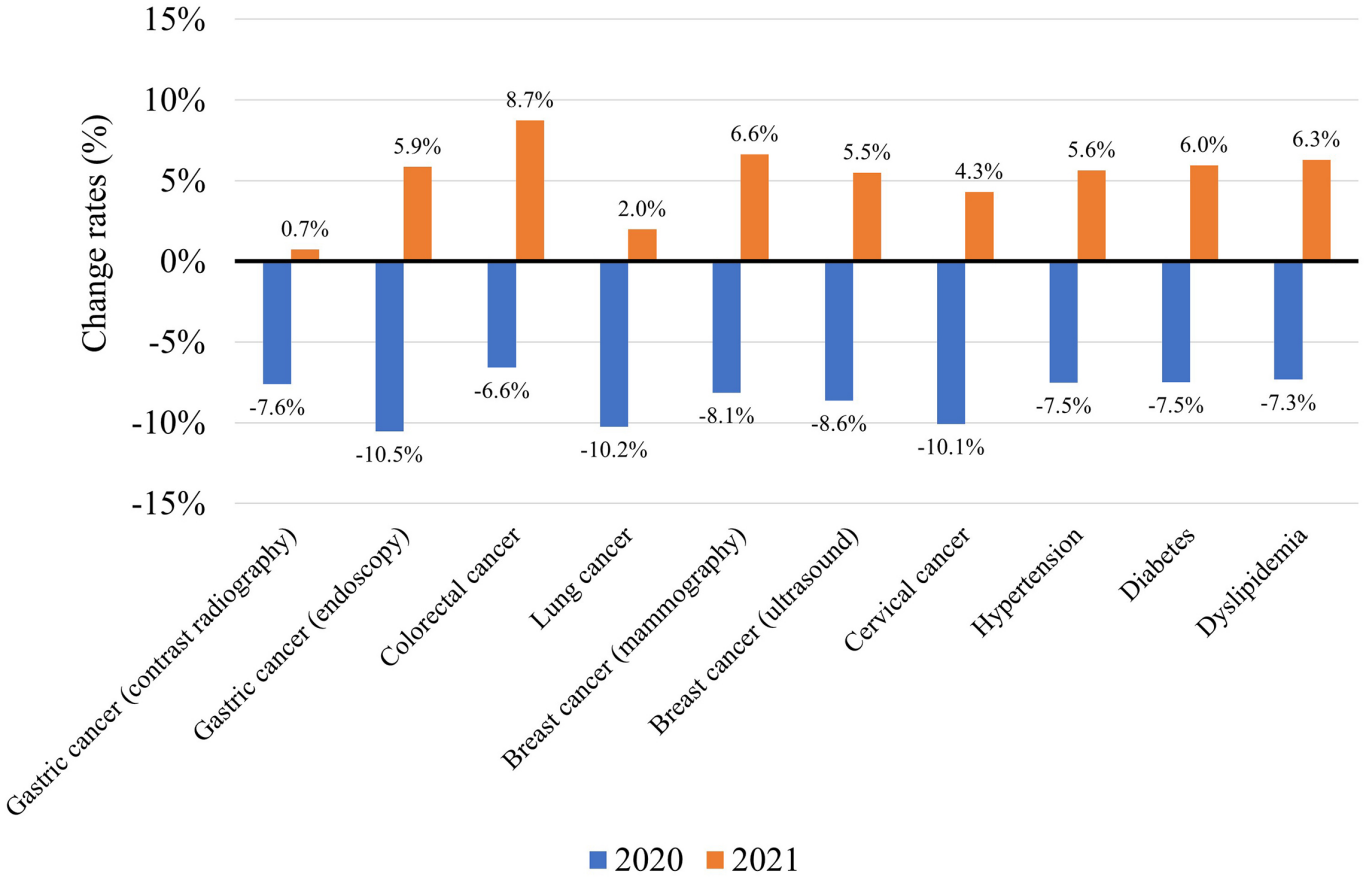
Change rates in the total number of screenings in 2020 and 2021 compared with that in 2019 (n = 371).

The total number of examinees undergoing screenings for cancer, hypertension, diabetes, and dyslipidemia in 371 facilities in 2020 and 2021 were compared with those in 2019. The total number of screenings for gastric cancer (contrast radiography or endoscopy), colorectal cancer (fecal occult blood), lung cancer (chest X-ray), breast cancer (mammography or ultrasound), cervical cancer (cytology), hypertension, diabetes, and dyslipidemia all decreased in 2020, as was previously reported ^(11)^. The number increased in 2021; however, except for colorectal cancer screenings, this increase was not enough to make up for the decrease in 2020 (Figure 2).

The proportions of examinees requiring follow-up visits among those undergoing screenings in 2020 and 2021 were compared with those in 2019 for each facility. As we previously reported ^(11)^, the proportions decreased in screenings for gastric and lung cancer in 2020, whereas those for colorectal cancer, hypertension, and dyslipidemia increased. In 2021, the proportions decreased in screenings for gastric, colorectal, lung, and breast cancer (Table 3), whereas it increased only for hypertension.

**Table 3.** The Proportion of Examinees Requiring Follow-Up Visits between 2019 and 2021.

	Rate % in 2019, median (IQR)	Rate % in 2020, median (IQR)	Difference % (2020−2019), median (IQR)	*P* value^a^	Rate % in 2021, median (IQR)	Difference % (2021−2019), median (IQR)	*P* value^a^	Number of facilities
Gastric cancer (contrast radiography)	4.1 (2.1, 7.3)	3.4 (1.7, 6.4)	−0.34 (−1.34, 0.18)	<0.001	3.1 (1.7, 5.9)	−0.60 (−1.92, 0.08)	<0.001	356
Gastric cancer (endoscopy)	3.7 (1.8, 6.6)	3.5 (1.6, 6.0)	−0.23 (−0.95, 0.20)	<0.001	3.5 (1.8, 6.0)	−0.18 (−1.17, 0.56)	0.003	342
Colorectal cancer	5.5 (4.7, 6.4)	5.6 (4.8, 6.5)	+0.12 (−0.31, 0.54)	<0.001	5.4 (4.5, 6.1)	−0.13 (−0.54, 0.25)	<0.001	365
Lung cancer	1.5 (0.9, 2.4)	1.4 (0.8, 2.3)	−0.09 (−0.40, 0.15)	<0.001	1.3 (0.7, 2.2)	−0.14 (−0.52, 0.14)	<0.001	365
Breast cancer (mammography)	5.1 (3.1, 7.6)	5.0 (3.0, 7.4)	−0.11 (−1.24, 0.69)	0.238	4.8 (3.0, 7.0)	−0.29 (−1.61, 0.88)	0.028	362
Breast cancer (ultrasound)	3.1 (1.9, 5.2)	3.0 (1.7, 4.7)	−0.17 (−1.04, 0.59)	0.016	2.7 (1.6, 4.5)	−0.22 (−1.39, 0.57)	<0.001	309
Cervical cancer	1.9 (1.0, 3.4)	1.9 (1.1, 3.4)	+0.03 (−0.47, 0.50)	>0.99	2.0 (1.1, 3.6)	+0.02 (−0.62, 0.74)	>0.99	361
Hypertension	1.7 (0.7, 2.8)	1.9 (0.7, 3.1)	+0.10 (−0.04, 0.49)	<0.001	1.8 (0.7, 2.9)	+0.07 (−0.15, 0.39)	<0.001	353
Diabetes	1.5 (0.8, 2.8)	1.6 (0.8, 2.8)	0.00 (−0.15, 0.17)	0.226	1.4 (0.8, 2.6)	−0.02 (−0.31, 0.18)	0.134	354
Dyslipidemia	4.5 (2.3, 6.6)	4.5 (2.5, 7.1)	+0.04 (−0.29, 0.48)	0.044	4.6 (2.6, 6.9)	0.00 (−0.46, 0.65)	0.477	352

^a^ *P* values comparing the numbers in 2020 and 2021 to those in 2019 using the Friedman test with Holm correction

Adherence to follow-up visits in 2020 and 2021 was compared with that in 2019 in each facility. Adherence improved for cervical cancer, hypertension, and diabetes in 2020, and for lung, breast (ultrasound), and cervical cancer and hypertension, diabetes, and dyslipidemia in 2021 (Table 4).

**Table 4.** Adherence to Follow-Up Visits between 2019 and 2021.

	Rate % in 2019, median (IQR)	Rate % in 2020, median (IQR)	Difference % (2020−2019), median (IQR)	*P* value^a^	Rate % in 2021, median (IQR)	Difference % (2021−2019), median (IQR)	*P* value^a^	Number of facilities
Gastric cancer (contrast radiography)	53.2 (39.5, 65.5)	54.9 (40.3, 66.7)	−0.2 (−5.6, 4.7)	0.669	56.5 (40.0, 68.5)	+0.7 (−5.8, 9.9)	0.248	300
Gastric cancer (endoscopy)	84.9 (63.8, 97.8)	84.6 (64.2, 98.0)	0.0 (−4.2, 2.9)	0.906	86.8 (64.7, 98.5)	0.0 (−4.1, 3.8)	0.939	295
Colorectal cancer	51.8 (39.9, 63.6)	52.8 (41.3, 62.8)	+0.2 (−4, 4.1)	0.648	53.2 (41.7, 64.6)	0.0 (−4.3, 5.0)	0.648	316
Lung cancer	66.3 (49.2, 80.4)	66.7 (51.2, 79.3)	+1.0 (−4.7, 6.6)	0.068	69.1 (53.5, 80.0)	+1.3 (−5.0, 8.0)	0.032	321
Breast cancer (mammography)	80.5 (60.1, 89.5)	81.3 (63.6, 89.0)	0.0 (−4.9, 6.2)	0.753	81.1 (64.5, 89.5)	0.0 (−6.1, 6.7)	>0.99	313
Breast cancer (ultrasound)	76.1 (55.7, 89.4)	79.7 (60.0, 90.7)	+0.2 (−5.6, 9.3)	0.096	80.2 (58.9, 91.3)	+1.4 (−4.8, 11.4)	0.014	248
Cervical cancer	71.6 (53.0, 83.7)	75.0 (56.9, 86.4)	+1.3 (−4.8, 9.9)	0.005	76.9 (58.1, 89.1)	+1.8 (−3.7, 11.7)	<0.001	299
Hypertension	30.5 (14.1, 43.9)	34.5 (17.2, 48.7)	+0.8 (−1.6, 7.5)	<0.001	37.1 (19.7, 52.4)	+1.8 (−2.3, 10.0)	<0.001	258
Diabetes	37.2 (19.2, 54.5)	39.6 (20.2, 56.1)	+1.1 (−2.5, 7.3)	0.002	40.3 (20.5, 59.9)	+1.7 (−1.9, 8.8)	<0.001	274
Dyslipidemia	29.6 (15.7, 43.5)	31.1 (15.7, 43.5)	+0.1 (−3.5, 3.1)	0.566	31.7 (16.7, 45.8)	+1.2 (−2.7, 6.5)	0.013	271

^a^ *P* values comparing the numbers in 2020 and 2021 to those in 2019 using the Friedman test with Holm correction

**Figure 3.**
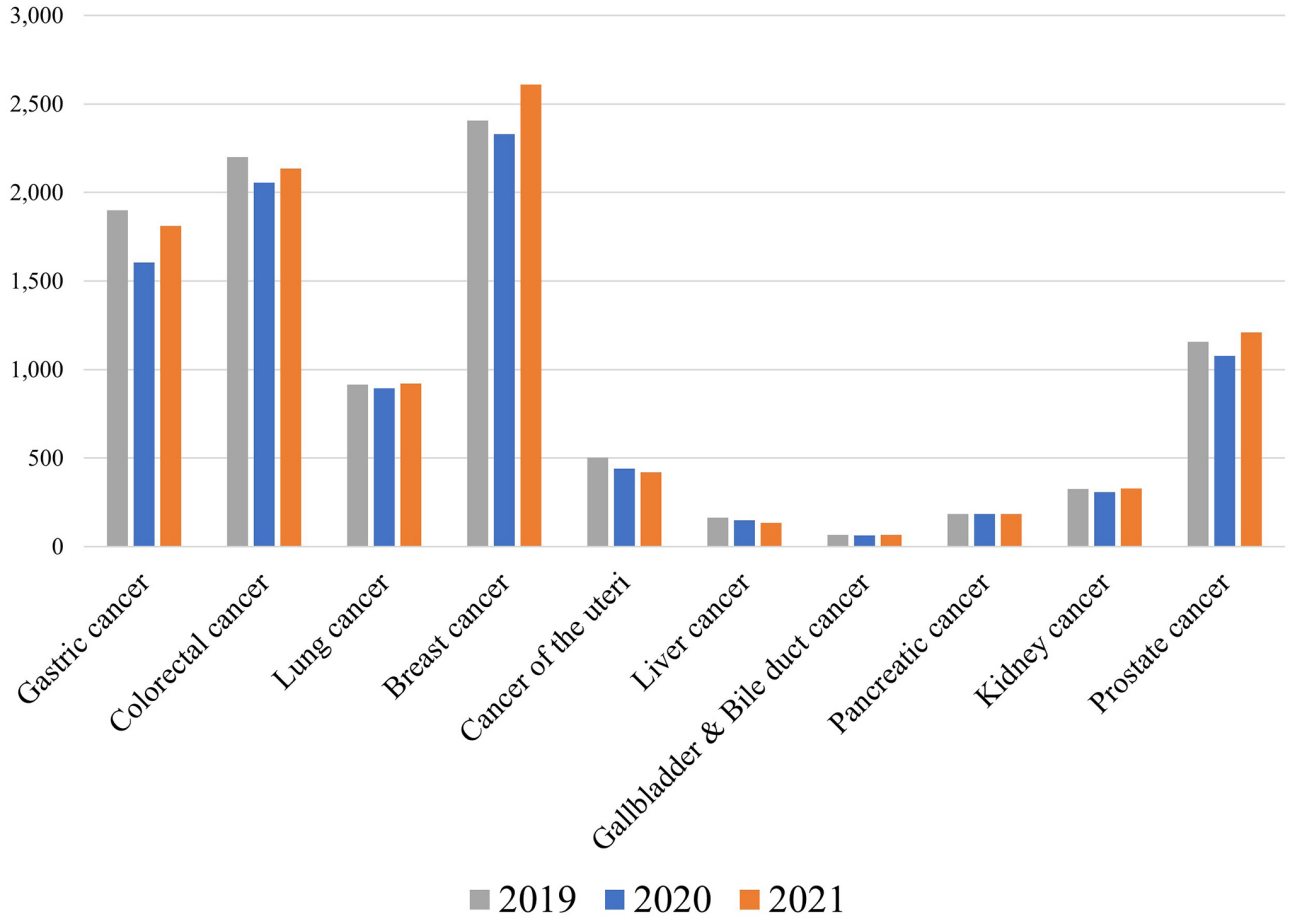
Total number of cancer cases found in 310 facilities.

The number of cancer cases found was reported by 310 facilities, although these numbers are likely to have been underreported as the facilities relied on the examinees’ self-reports. The total number of cancer cases found by health check-ups in 310 facilities decreased in 2020 compared with that of 2019 for most cancer types, presumably due to the decreased number of check-ups (Figure 3). In 2021, the number of cancer cases decreased for gastric, colorectal, uterine and cervical (cancer of the uteri), and liver cancer, whereas it increased for breast and prostate cancer. As prostate cancer screenings based on prostate-specific antigen tests are mainly performed as optional tests in Ningen Dock, whose number recovered to the prepandemic level in 2021 (Figure 1), it is likely that the number of examinees undergoing prostate cancer screenings also recovered in 2021, although the exact number is unknown.

### Precautions against COVID-19

Facilities were asked about their precautions against COVID-19 when performing in-facility check-ups. Over 90% of the facilities reported applying protocols such as wearing masks, handwashing/hand disinfection, checking body temperature and health conditions of the examinees, proper ventilation, disinfection of high-touch surfaces, and establishing procedures for examinees who were judged inappropriate to undergo check-ups. Over 80% of the facilities reported layout changes to avoid overcrowding, limiting, or rescheduling appointments to prevent overcrowding, recommendations to wear unwoven masks, and installing partitions (Supplementary Table 1).

Furthermore, facilities were asked whether they had made specific recommendations to their employees regarding precautions against COVID-19. Of the 838 facilities, 805 (96.1%) requested that their employees refrain from conversation while eating, 485 (57.9%) requested them to refrain from having meals with people who did not live with them, and 639 (76.3%) requested them to refrain from traveling after the first State of Emergency (April-May 2020) was lifted (Supplementary Table 2). Even in 2022, 329 (39.3%) and 301 (35.9%) of the facilities requested their employees to refrain from having meals with people who did not live with them and to refrain from traveling, respectively. In 803 (95.8%) facilities, employees experiencing a fever were obligated to undergo COVID-19 tests, and in 500 (59.7%) facilities, employees were not allowed to work for certain periods, even when their COVID-19 tests were negative. Regarding vaccination, 752 (89.7%) facilities encouraged their employees to get vaccinated, and 658 (78.5%) recommended the fourth and subsequent vaccination doses.

When recommendations to employees were compared according to facility type, those annexed to hospitals were more likely to make these recommendations to the employees than those not annexed to hospitals (Supplementary Table 2). Conversely, few differences were found in COVID-19 precautions when performing in-facility check-ups (Supplementary Table 1).

### Absence of employees for COVID-19-related reasons

**Figure 4.**
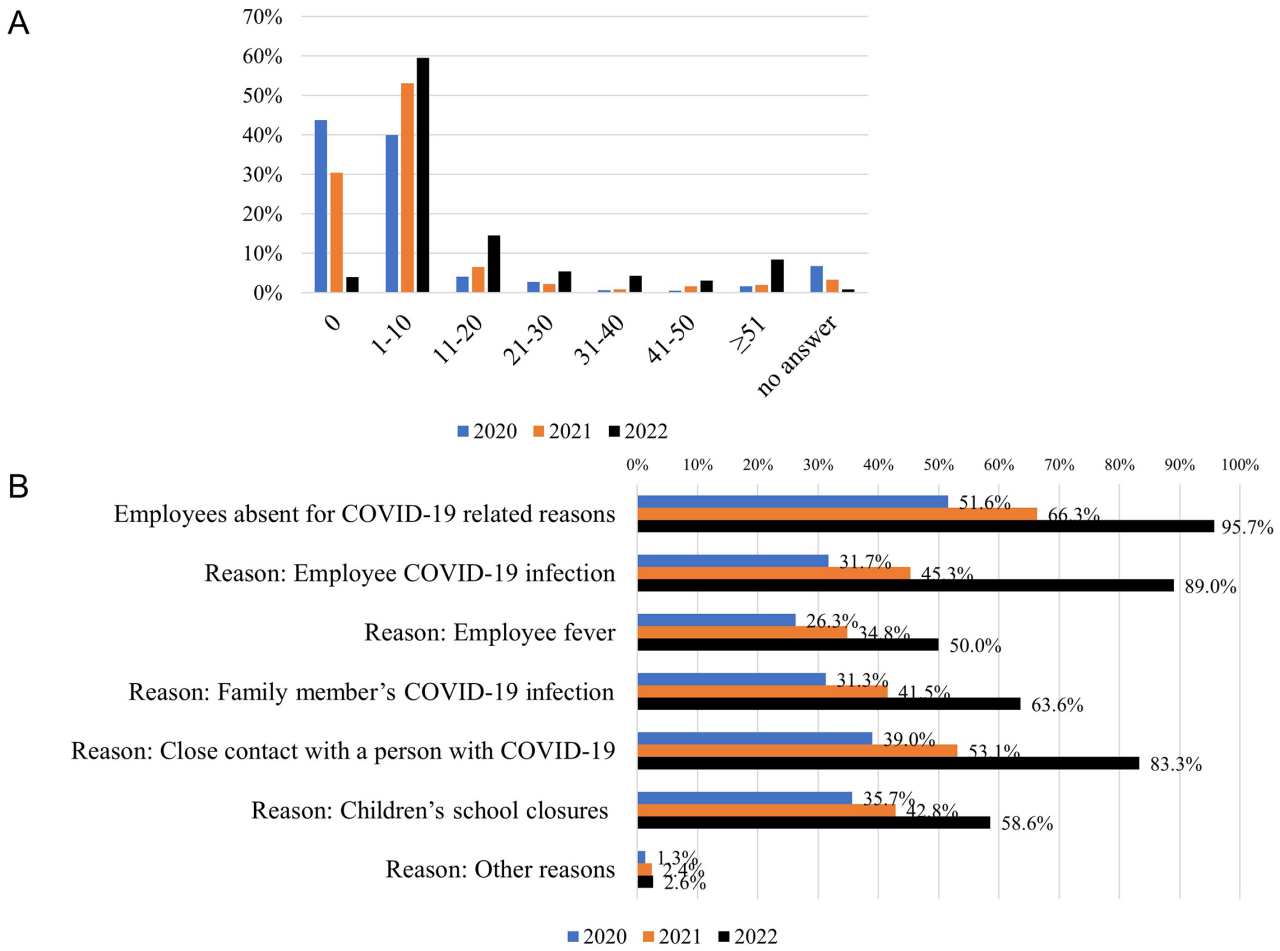
COVID-19-related employee absence. (A) Number of employees absent for COVID-19-related reasons in 2020-2022 (B) Main reasons for absence in each year (multiple choice).

Facilities provided the number of employees absent for COVID-19-related reasons each year between 2020 and 2022, as well as the main reasons for these absences. Employee absence was reported by 432 (51.6%), 556 (66.3%), and 802 (95.7%) facilities in 2020, 2021, and 2022, respectively. The number of absent employees increased over time (Figure 4A). Close contact with an infected person (39.0%) and children’s school closures (35.7%) were the most frequent reasons for employee absence in 2020, while employee infection with COVID-19 (89.0%) was the most frequent reason in 2022, followed by close contact with an infected person (83.3%) (Figure 4B).

### Characteristics of the facilities according to negative financial impact in 2022

Characteristics of the facilities according to whether they had a negative financial impact in 2022 are shown in Supplementary Table 3. Those that experienced a negative impact were more likely to be annexed to hospitals, be public institutions, have a larger number of employees, and have a lower percentage of mandatory check-ups in 2019. Among the facilities providing the number of examinees, the median percent change in the number of check-ups in 2022 compared with that of 2019 was −3.2% (IQR: −9.3 to 3.4%; P < 0.001) for facilities with a negative financial impact in 2022 (n = 299), whereas it was +1.5% (IQR: −3.5 to 11.6%; P = 0.061) for those without a negative financial impact (n = 124).

Multivariate analyses were performed to identify the factors associated with a negative financial impact in 2022. Among the 838 facilities that responded to Part I, being annexed to hospitals, being located in ordinance-designated cities/special wards in Tokyo, having a larger number of employees, and not accepting admission of patients with COVID-19 were significantly associated with a negative financial impact in 2022 (Table 5). In a model for the facilities responding to both Parts I and II, not providing COVID-19 vaccination services and a lower proportion of mandatory check-ups in 2019 were also found to be associated with a negative financial impact.

**Table 5.** Multivariate Logistic Regression Model for the Negative Financial Impact in 2022.

		Univariate		Multivariate Model 1 ^a^		Multivariate Model 2 ^b^	
		Odds ratio (95% CI)	*P* value	Odds ratio (95% CI)	*P* value	Odds ratio (95% CI)	*P* value
(Intercept)		-		0.87 (0.47, 1.59)	0.645	1.19 (0.43, 3.28)	0.743
Type of facility	Dedicated to healthcare check-ups/annexed to clinics	Ref		Ref		Ref	
	Annexed to hospitals	1.66 (1.23, 2.24)	<0.001	3.57 (2.03, 6.27)	<0.001	4.02 (1.77, 9.13)	<0.001
Area	Kanto	Ref		Ref		Ref	
	Hokkaido	0.51 (0.21, 1.28)	0.151	0.47 (0.18, 1.22)	0.122	0.37 (0.09, 1.62)	0.189
	Tohoku	1.77 (0.81, 3.86)	0.153	1.89 (0.85, 4.23)	0.120	1.62 (0.53, 4.98)	0.398
	Chubu	1.31 (0.85, 2.03)	0.219	1.25 (0.79, 1.99)	0.334	1.41 (0.69, 2.86)	0.343
	Kansai	0.72 (0.47, 1.09)	0.117	0.77 (0.50, 1.19)	0.235	0.75 (0.40, 1.39)	0.358
	Chugoku	1.08 (0.59, 1.97)	0.800	1.09 (0.59, 2.04)	0.778	1.07 (0.45, 2.51)	0.881
	Shikoku	0.42 (0.17, 1.05)	0.063	0.44 (0.17, 1.16)	0.098	0.61 (0.17, 2.12)	0.434
	Kyushu	0.85 (0.49, 1.46)	0.555	0.88 (0.50, 1.56)	0.671	0.88 (0.37, 2.10)	0.774
	Okinawa	2.31 (0.49, 10.9)	0.290	2.59 (0.52, 12.97)	0.247	4.27 (0.47, 38.58)	0.196
Location of facilities	Ordinance-designated cities or special wards in Tokyo vs. others	1.29 (0.95, 1.76)	0.102	1.52 (1.07, 2.15)	0.020	1.40 (0.85, 2.32)	0.187
Public or private	Public	1.56 (1.08, 2.26)	0.019	1.42 (0.93, 2.17)	0.105	1.02 (0.56, 1.87)	0.944
Number of employees	1-10	Ref		Ref		Ref	
	11-50	1.91 (1.29, 2.83)	0.001	1.73 (1.13, 2.64)	0.011	2.20 (1.03, 4.67)	0.041
	>50	2.01 (1.22, 3.30)	0.006	2.05 (1.17, 3.61)	0.012	2.65 (0.99, 7.09)	0.053
Accepting admission of patients with COVID-19	Yes vs. no	1.19 (0.89, 1.60)	0.238	0.59 (0.35, 0.98)	0.042	0.86 (0.42, 1.76)	0.671
Outpatient fever clinic	Yes vs. no	1.07 (0.79, 1.45)	0.646	0.88 (0.55, 1.41)	0.591	0.71 (0.35, 1.41)	0.327
Providing COVID-19 vaccination services	Yes vs. no	0.76 (0.53, 1.10)	0.142	0.65 (0.42, 1.00)	0.051	0.51 (0.27, 0.96)	0.037
COVID-19 PCR center	Yes vs. no	0.80 (0.55, 1.17)	0.249	0.75 (0.50, 1.13)	0.169	1.10 (0.58, 2.06)	0.777
Providing help to other departments	Yes vs. no	1.22 (0.91, 1.64)	0.192	1.13 (0.81, 1.57)	0.474	1.07 (0.67, 1.73)	0.767
Total number of check-ups in 2019	<10000	Ref				Ref	
	10000-19999	1.31 (0.78, 2.19)	0.310			0.94 (0.50, 1.75)	0.839
	20000-39999	1.42 (0.80, 2.54)	0.233			1.36 (0.65, 2.85)	0.418
	≥40000	1.50 (0.84, 2.67)	0.170			1.25 (0.52, 3.02)	0.612
Proportion of mandatory check-ups^c^ in 2019	<34%	Ref				Ref	
	≥34%	0.58 (0.38, 0.89)	0.013			0.55 (0.34, 0.90)	0.016

^a^ Model for the facilities that responded to Part I ^b^ Model for the facilities that responded to Parts I and II ^c^ Proportion of “Check-ups based on Industrial Safety and Health Act” and “Check-ups for prevention of lifestyle-related diseases”

## Discussion

We have previously reported that although the number of examinees undergoing mandatory check-ups recovered by 2021 from the initial decline caused by the COVID-19 pandemic, the number of examinees undergoing nonmandatory check-ups remained low in 2021 based on our nationwide questionnaire survey targeting healthcare facilities ^(11)^. In this follow-up survey, we report that this tendency continued even in 2022, raising concerns about delays in the diagnosis and treatment for cancer and diabetes, especially in populations ineligible for mandatory check-ups, such as those who are self-employed, part-time workers, retired, unemployed, or dependents. Moreover, approximately 60% of the facilities reported negative financial impacts on the management of health check-ups, even in 2022.

The exact reasons for the incomplete recovery in the number of health check-ups in 2022 are yet to be elucidated; however, several potential reasons can be considered. It is possible that people who had been hesitant to undergo check-ups due to the pandemic still had difficulty returning to check-ups, or that the unprecedented numbers of COVID-19 cases in 2022 deterred people from visiting health check-up facilities. Some facilities may have also had difficulties in accepting examinees due to employees getting infected with COVID-19, resulting in their absence. Further studies are warranted to determine whether the numbers will fully recover to preCOVID-19 levels as the pandemic calms down, and to ascertain the long-term impacts of delayed recovery.

Although the number of screenings for cancer and diabetes increased in 2021 compared with that in 2019, this increase was insufficient to make up for missed screenings in 2020. The proportion of examinees requiring follow-up visits among those who underwent screenings decreased for gastric, colorectal, lung, and breast cancer in 2021 compared with that in 2019. These results must be interpreted with caution, as the proportion of examinees requiring follow-up visits tends to decrease as screening technologies improve over time, regardless of increases in incidence rates. Considering that the number of examinees undergoing mandatory check-ups for full-time employees increased but that those undergoing nonmandatory cancer screenings by local governments remained low in 2021 and 2022, it is also possible that changes in the examinees’ age structure (e.g., older people with a higher risk of cancer were less likely to have undergone cancer screenings in 2021 than in preCOVID-19 years) might have contributed to these results. Adherence to follow-up visits among those who were required to have them varied depending on the type of screening. Adherence was higher for cancer screenings than for diabetes, hypertension, or dyslipidemia screenings, which is consistent with previous reports ^(11), (14), (15), (16)^. Adherence improved for some cancer screenings, as well as for diabetes, hypertension, and dyslipidemia screenings in 2021, suggesting that those who underwent screenings in 2021 were more likely to have higher health literacy.

Globally, studies have reported that cancer screening programs sharply declined during the early stages of the pandemic but rebounded by the summer of 2020 ^(17), (18), (19)^. However, more recent studies with longer follow-up times revealed that decreases in cancer screenings did not resolve in 2021. Studies from the US reported that in 2021, screening prevalence for breast, cervical, and prostate cancer continued to be lower than the preCOVID-19 level ^(5), (6)^. Another study reported that breast and lung cancer screening rates among Medicare enrollees were below the expected rates between March 2021 and February 2022 ^(20)^. Moreover, a study from the UK reported that diabetes monitoring and screening between January 2019 and December 2021, as evaluated by the volume of HbA1c testing, dropped three times in April-June 2020 during the first lockdown, in January 2021 during the second lockdown, and in October 2021, coinciding with the national shortage of blood specimen tubes, and did not achieve the expected levels even during the intervening periods ^(7)^. Substantial increases in the number of avoidable cancer deaths are expected owing to the delays in diagnosis and subsequent treatments ^(3)^, and an increase in the proportion of advanced lung cancer has been reported ^(21), (22)^. Improving screening rates to preCOVID-19 levels is a pressing task to reduce avoidable deaths in the future.

Previous studies suggested that hospitals in the US experienced financial strain owing to increased costs related to COVID-19 and the lost revenue from decreased outpatient visits, elective surgeries, and procedures ^(23), (24)^. In this study, 61.7% of the facilities reported that they experienced a negative financial impact on the management of health check-ups even in 2022 due to the pandemic, and the number of examinees undergoing health check-ups decreased in these facilities in 2022 compared with that in 2019. Factors associated with a negative financial impact in 2022 included being annexed to hospitals, having a larger number of employees, being located in big cities, and having a lower proportion of mandatory check-ups in 2019. Facilities annexed to hospitals may prioritize managing hospital wards rather than health check-ups, which may contribute to delays in the recovery of the number of examinees.

Our results also revealed that employees in health check-up facilities were subject to strict rules and had to make substantial sacrifices in their private lives (Supplementary Table 2). Even in 2022, more than one-third of the facilities requested their employees to refrain from having meals with people who did not live with them or from traveling. Notably, facilities annexed to hospitals imposed more strict recommendations on their employees than those not annexed to hospitals, which is probably because those working in facilities annexed to hospitals had more contact opportunities with patients susceptible to COVID-19 infection, and clusters in these facilities would have serious consequences. Indeed, 66.2% of the facilities annexed to hospitals reported that their employees had to help other departments within the facilities.

### Strength and limitations

The strength of this study is that it is a large-scale nationwide survey conducted in Japan with a response rate of >60%. Moreover, we compared the number of examinees undergoing health check-ups between a preCOVID-19 year and the first, second, and third years of the pandemic. To the best of our knowledge, this is the first large-scale study to investigate the financial impact of the pandemic on the management of health check-up facilities. Furthermore, no previous studies have identified the burden experienced by employees in health check-up facilities regarding COVID-19 precautions.

This study had several limitations. First, as Japan’s health check-up system is unique, the results may not be generalizable to other areas. However, our results were consistent with reports from other countries, which found that cancer and diabetes screenings did not fully recover to the preCOVID-19 level after the initial decline and rebound in 2020. Second, no information on the examinees’ demographics, such as age and sex, was available. Third, the number of cancer cases found may have been underreported, as the health check-up facilities rely on the examinees to self-report the results of their follow-up visits.

### Conclusion

The number of examinees undergoing nonmandatory check-ups remained low compared with that of the preCOVID-19 period, even in 2022. The total number of cancer screenings recovered to the preCOVID-19 level in 2021; however, it was insufficient to make up for missed screenings in 2020. Public health efforts are required to encourage people to return to cancer and diabetes screenings.

## Article Information

### Conflicts of Interest

SY, AO, and TK are members of the Department of Prevention of Diabetes and Lifestyle-related Diseases, which is a cooperative program between The University of Tokyo and Asahi Mutual Life Insurance Company. TA, SN, TY, YA, TA, and MN declare no competing interests.

### Sources of Funding

This work was supported by MHLW Research on Emerging and Re-emerging Infectious Diseases and Immunization (Program Grant Numbers JPMH21HA2011 and JPMH23HA2011). The funding organization has no role in the study’s design, analysis, interpretation of data, or in writing the manuscript.

### Acknowledgement

We would like to thank all of the participating facilities for taking the time to respond to the questionnaire. We thank Ms. Emi Yoshikawa and Mr. Shoji Negishi of Japan Society of Ningen Dock for their support.

### Author Contributions

SY, TA, AO, SN, MN, and TK designed the study. TA and SN acquired data. SY analyzed the data. SY and TK wrote the first draft of the manuscript. All authors contributed to the interpretation of data, and reviewed, revised, and approved the final manuscript. SY and TA contributed equally as the first authors. SY and TK contributed equally as corresponding authors.

### Approval by Institutional Review Board (IRB)

This study was approved by the institutional review board of the Graduate School of Medicine at the University of Tokyo (2018030NI) and was performed following the Declaration of Helsinki. The need for informed consent was waived because this was a facility-based survey containing no personal information.

## Supplement

Supplementary Materials
