# Supplementary material for: Impact of the COVID-19 Pandemic on Health Check-ups in 2021 and 2022: A Nationwide Follow-up Survey of Healthcare Facilities in Japan Society of Ningen Dock
Source: JMA J. 2023 Dec 27;7(1):94–105. doi: 10.31662/jmaj.2023-0126 (PMC10834170; doi:10.31662/jmaj.2023-0126)
Supplement: Supplementary Materials [file 2433-3298-7-1-0094-s001.pdf]

Impact of the COVID-19 pandemic on health check-ups in 2021 and 2022: A nationwide follow-up survey of healthcare facilities in Japan Society of Ningen Dock

Supplementary Materials

Supplementary Table 1. Precautions against COVID-19 when performing in-facility check-ups

	Total (n=838)	Dedicated to check-ups/Annexed to clinics (n=320)	Annexed to hospitals (n=518)	*P* value
Wearing masks (employees and examinees)	835 (99.6)	318 (99.4)	517 (99.8)	0.673
Handwashing/hand disinfection	834 (99.5)	318 (99.4)	516 (99.6)	>0.99
Sufficient ventilation	816 (97.4)	314 (98.1)	502 (96.9)	0.398
Checking body temperature and health conditions of the examinees	823 (98.2)	313 (97.8)	510 (98.5)	0.679
Limiting or rescheduling appointments to prevent overcrowding	749 (89.4)	291 (90.9)	458 (88.4)	0.301
Disinfection of high-touch surfaces	816 (97.4)	312 (97.5)	504 (97.3)	>0.99
Establishing procedures for examinees who are judged inappropriate to undergo check-ups	813 (97.0)	306 (95.6)	507 (97.9)	0.098
Preparation of procedures for dealing with suspected cases of COVID-19 infection on chest X-rays	511 (61.0)	213 (66.6)	298 (57.5)	0.011
Questionnaires on COVID-19 (e.g. behavioral history, contact history)	551 (65.8)	173 (54.1)	378 (73.0)	<0.001
Layout changes to avoid overcrowding	752 (89.7)	287 (89.7)	465 (89.8)	>0.99
Installing partitions	699 (83.4)	294 (91.9)	405 (78.2)	<0.001
Asking examinees to wear unwoven masks	747 (89.1)	285 (89.1)	462 (89.2)	>0.99
Restrictions of visits from examinees from COVID-19-prevalent areas	388 (46.3)	126 (39.4)	262 (50.6)	0.002
COVID-19 antigen tests	125 (14.9)	45 (14.1)	80 (15.4)	0.656
Removal of newspapers, magazines, and blankets	586 (69.9)	235 (73.4)	351 (67.8)	0.096
Recommendation that examinees refrain from unnecessary conversation	505 (60.3)	210 (65.6)	295 (56.9)	0.015
Stopping providing food/Asking examinees to refrain from unnecessary conversation while eating	329 (39.3)	104 (32.5)	225 (43.4)	0.002

Supplementary Table 2. Recommendations to health check-up facility employees as precautions against COVID-19

	Total (n=838)	Dedicated to check-ups/Annexed to clinics (n=320)	Annexed to hospitals (n=518)	*P* value
Refrain from conversations while eating	805 (96.1)	297 (92.8)	508 (98.1)	<0.001
Refrain from conversations without wearing masks	763 (91.1)	284 (88.8)	479 (92.5)	0.087
Refrain from having meals with people who do not live with them	485 (57.9)	150 (46.9)	335 (64.7)	<0.001
in 2020	459 (54.8)	142 (44.4)	317 (61.2)	<0.001
in 2021	423 (50.5)	127 (39.7)	296 (57.1)	<0.001
in 2022	329 (39.3)	94 (29.4)	235 (45.4)	<0.001
Wear unwoven masks	823 (98.2)	312 (97.5)	511 (98.6)	0.342
Use either face shields, eye shields, or goggles	490 (58.5)	160 (50.0)	330 (63.7)	<0.001
Refrain from travelling	639 (76.3)	213 (66.6)	426 (82.2)	<0.001
in 2020 (after the first State of Emergency was lifted)	600 (71.6)	201 (62.8)	399 (77.0)	<0.001
in 2021	532 (63.5)	166 (51.9)	366 (70.7)	<0.001
in 2022	301 (35.9)	78 (24.4)	223 (43.1)	<0.001
Take COVID-19 tests for fever or cold symptoms	803 (95.8)	299 (93.4)	504 (97.3)	0.011
Avoid work for certain periods with fever or cold symptoms, even if COVID-19 tests were negative	500 (59.7)	180 (56.3)	320 (61.8)	0.131
Get vaccinated against COVID-19	752 (89.7)	271 (84.7)	481 (92.9)	<0.001
Three doses of vaccination	60 (7.2)	31 (9.7)	29 (5.6)	0.013
Fourth and subsequent doses of vaccination	658 (78.5)	228 (71.3)	430 (83.0)	

Supplementary Table 3. Basic facility characteristics according to the negative financial impact of COVID-19 in 2022

		Total (n=838)	No negative impact (n=273)	Negative impact (n=517)	*P* value
Type of facility	Dedicated to healthcare check-ups/annexed to clinics	320 (38.2)	126 (46.2)	176 (34.0)	0.001
	Annexed to hospitals	518 (61.8)	147 (53.8)	341 (66.0)	
Area	Kanto	301 (35.9)	97 (35.5)	189 (36.6)	0.042
	Hokkaido	21 (2.5)	10 (3.7)	10 (1.9)	
	Tohoku	44 (5.3)	9 (3.3)	31 (6.0)	
	Chubu	157 (18.7)	41 (15.0)	105 (20.3)	
	Kansai	148 (17.7)	58 (21.2)	81 (15.7)	
	Chugoku	60 (7.2)	19 (7.0)	40 (7.7)	
	Shikoku	23 (2.7)	11 (4.0)	9 (1.7)	
	Kyushu	73 (8.7)	26 (9.5)	43 (8.3)	
	Okinawa	11 (1.3)	2 (0.7)	9 (1.7)	
Location of facilities	Ordinance-designated cities or special wards in Tokyo	310 (37.0)	90 (33.0)	201 (38.9)	0.119
Public or private	Public	190 (22.7)	48 (17.6)	129 (25.0)	0.023
Number of employees	1–10	140 (16.7)	61 (22.3)	67 (13.0)	0.029
	11–20	237 (28.3)	74 (27.1)	147 (28.4)	
	21–30	163 (19.5)	47 (17.2)	112 (21.7)	
	31–40	86 (10.3)	26 (9.5)	55 (10.6)	
	41–50	66 (7.9)	21 (7.7)	39 (7.5)	
	51 or more	146 (17.4)	44 (16.1)	97 (18.8)	
Accepting admission of COVID-19 patients	Yes	419 (50.0)	130 (47.6)	269 (52.0)	0.269
Outpatient fever clinic	Yes	518 (61.8)	166 (60.8)	323 (62.5)	0.702
Providing COVID-19 vaccination services	Yes	650 (77.6)	222 (81.3)	397 (76.8)	0.168
COVID-19 PCR center	Yes	149 (17.8)	55 (20.1)	87 (16.8)	0.290
Providing staffs to other departments in the facility	Yes	482 (57.5)	150 (54.9)	309 (59.8)	0.218
Providing help to other departments for COVID-19-related care	Yes	359 (42.8)	110 (40.3)	233 (45.1)	0.225
		(n=448)	(n=124)	(n=299)	

Total number of check-ups in 2019	Median (Q1,Q3)	13189 (6524, 31302)	12709 (5473, 26870)	14208 (7373, 32122)	0.157
	<10000	176 (39.3)	67 (54.0)	130 (43.5)	0.694
	10000–19999	106 (23.7)	30 (24.2)	76 (25.4)	
	20000–39999	79 (17.6)	21 (16.9)	58 (19.4)	
	≥40000	87 (19.4)	21 (16.9)	61 (20.4)	
Percentage of mandatory check-ups[a] in 2019	Median (Q1,Q3)	33.0 (16.9, 53.7)	40.4 (21.9, 60.7)	31.2 (15.4, 50.2)	0.004
	<34%	229 (51.1)	52 (41.9)	166 (55.5)	0.015
	≥34%	219 (48.9)	72 (58.1)	133 (44.5)	
Percent change in the number of check-ups in 2022 compared to 2019	Median (Q1,Q3)	-1.4 (-7.4, 5.7)	1.5 (-3.5, 11.6)	-3.2 (-9.3, 3.4)	<0.001

[a] Percentage of “Check-ups based on Industrial Safety and Health Act” and “Check-ups for prevention of lifestyle-related diseases”

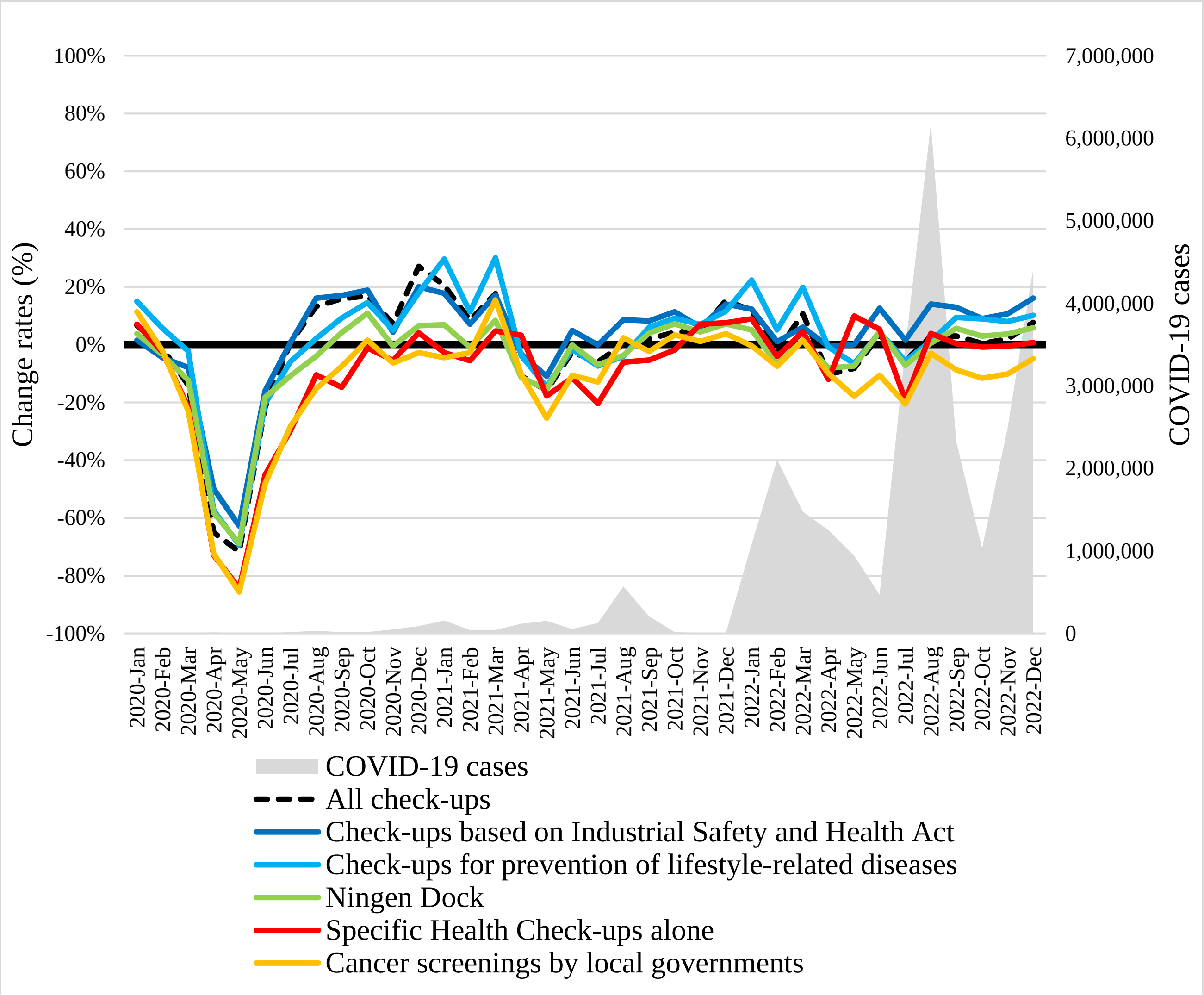

Supplementary Figure 1. Change rates in the total overall monthly numbers of check-ups and those by type between 2020 and 2022, as compared to the same months in 2019 (n=403). Monthly numbers of COVID-19 cases are also shown.
